# Use of capillary Western immunoassay (Wes) for quantification of dystrophin levels in skeletal muscle of healthy controls and individuals with Becker and Duchenne muscular dystrophy

**DOI:** 10.1371/journal.pone.0195850

**Published:** 2018-04-11

**Authors:** Chantal Beekman, Anneke A. Janson, Aabed Baghat, Judith C. van Deutekom, Nicole A. Datson

**Affiliations:** BioMarin Nederland BV, Leiden, The Netherlands; Rutgers University Newark, UNITED STATES

## Abstract

Duchenne muscular dystrophy (DMD) is a neuromuscular disease characterized by progressive weakness of the skeletal and cardiac muscles. This X-linked disorder is caused by open reading frame disrupting mutations in the DMD gene, resulting in strong reduction or complete absence of dystrophin protein. In order to use dystrophin as a supportive or even surrogate biomarker in clinical studies on investigational drugs aiming at correcting the primary cause of the disease, the ability to reliably quantify dystrophin expression in muscle biopsies of DMD patients pre- and post-treatment is essential. Here we demonstrate the application of the ProteinSimple capillary immunoassay (Wes) method, a gel- and blot-free method requiring less sample, antibody and time to run than conventional Western blot assay. We optimized dystrophin quantification by Wes using 2 different antibodies and found it to be highly sensitive, reproducible and quantitative over a large dynamic range. Using a healthy control muscle sample as a reference and α-actinin as a protein loading/muscle content control, a panel of skeletal muscle samples consisting of 31 healthy controls, 25 Becker Muscle dystrophy (BMD) and 17 DMD samples was subjected to Wes analysis. In healthy controls dystrophin levels varied 3 to 5-fold between the highest and lowest muscle samples, with the reference sample representing the average of all 31 samples. In BMD muscle samples dystrophin levels ranged from 10% to 90%, with an average of 33% of the healthy muscle average, while for the DMD samples the average dystrophin level was 1.3%, ranging from 0.7% to 7% of the healthy muscle average. In conclusion, Wes is a suitable, efficient and reliable method for quantification of dystrophin expression as a biomarker in DMD clinical drug development.

## Introduction

Duchenne muscular dystrophy (DMD) is a neuromuscular disease that affects 1 in 5000–6000 newborn boys [1,2] and is characterized by progressive weakness of the skeletal and cardiac muscles, respiratory failure and death in early adulthood [2,3]. This X-linked disorder is caused by mutations in the DMD gene which codes for dystrophin, a large 427 kDa protein critical for sarcolemmal integrity and with an important role in intracellular signaling [4,5]. The dystrophin protein links the intracellular cytoskeleton network (actin) to transmembrane components of the dystrophin-associated glycoprotein complex (DGC), including dystroglycan, sarcoglycans and sarcospan [6]. This function requires intact N-terminal actin-binding and C-terminal DGC-binding domains, while the central rod domain (spectrin-like repeats) seems to be less important. The mutations in DMD disrupt the open reading frame and therefore result in prematurely truncated and instable dystrophin variants lacking the C-terminus, giving rise to strongly reduced levels or absence of dystrophin. Mutations that conserve the reading frame lead to a shorter dystrophin protein typically lacking part of the central rod domain region and underlie the milder Becker muscular dystrophy (BMD) [7–9].

Currently, several potential therapies are being explored and developed to restore dystrophin in muscle of DMD patients, including gene therapy, stop codon read through, exon skip inducing antisense oligonucleotides (AONs) and CRISPR/Cas9 technology. To be able to monitor the efficiency of these therapies, reliable quantification of dystrophin expression as a supportive biomarker is essential. The use of dystrophin as a surrogate biomarker is under debate as it remains unresolved how much dystrophin is required for a clinically relevant improvement of muscle function. Several attempts have been made to correlate dystrophin levels in BMD patients to clinical severity, but this is hampered by the highly variable nature of the mutations in BMD, affecting different functional domains of dystrophin. BMD mutations shortening the central rod domain of dystrophin are generally correlated with a less severe phenotype, despite giving rise to a wide variety in dystrophin levels, while in-frame deletions affecting the N-terminal actin-binding domain generally result in a more severe disease course, indicating that not just levels of dystrophin but also the composition of the mutant dystrophin protein in BMD affect the clinical outcome ([10–13]. Genetic background and environmental factors are disease modifiers as well. Despite lack of an overall clear correlation between dystrophin levels and disease severity in BMD, it has been suggested that dystrophin levels below 10% of normal levels are associated with a more severe disease course, suggesting a threshold rather than a dose–effect relation, at least for mutations affecting the central rod domain region. Therefore, dystrophin restoring therapeutic approaches achieving around 10% of normal dystrophin levels may be sufficient to change a severe DMD phenotype to a milder BMD-like phenotype [13]. However, it must be noted that a DMD patient with restoration of a BMD-like dystrophin will never be completely comparable to a BMD patient who has had the protein expressed in all relevant tissues throughout development. Further adding to the complexity of the dystrophin biomarker discussion is the fact that dystrophin levels may vary between donors, different skeletal muscles, smooth muscle, diaphragm and cardiac muscle [14,15], questioning whether quantification of dystrophin in a small biopsy taken from a specific skeletal muscle is representative of the entire muscle compartment of the body and therefore of the full benefit of dystrophin-restoring therapies.

Besides these challenges, there is a lack of standardized methodologies for dystrophin quantification using a standard reference, indicated by regulatory authorities to be an obstacle to the advancement of the field [16]. Immunofluorescence analysis (IFA) and Western Blot (WB) are the most widely used methods for measuring dystrophin. In IFA dystrophin signal localised to the muscle fiber membranes can either be quantified or the number of positive fibers counted [17–19]. WB may be considered more quantitative than IFA since a calibration curve can be included, but has its own difficulties, including the large amount of protein required, technical challenges given the large size of dystrophin which is difficult to blot and the fact that it cannot discriminate dystrophin derived from revertant fibers or trace dystrophin. Other assays including reference standards, such as ELISA and Mass Spectrometry (MS), are also being developed, but have proven difficult (ELISA) [20], or require resources that are not available to every lab [20,21].

There is thus still a need for a fully quantitative and standardized method to accurately quantify dystrophin over a wide range of levels, preferably making use of a representative reference control to allow comparison between clinical studies in pre-treatment versus post-treatment samples. Full-length purified dystrophin protein that can serve as a reference standard is not currently available due to the large size and low expression of the dystrophin protein [22].

Here we demonstrate the application of the ProteinSimple capillary immunoassay (Wes) method for dystrophin detection. Wes has been shown to sensitively and reproducibly detect proteins of various sizes, while needing only small amounts of sample and taking much less time than WB [23,24]. In Wes, proteins are size separated in capillaries, where they are incubated with primary and (HRP-conjugated) secondary antibodies and finally with Luminol/peroxidase. The produced chemiluminescence is detected at multiple exposure times and automatically quantified by the Compass software. The chemiluminescent signal can be displayed as an electropherogram or as a virtual blot-like image. The electropherogram shows the intensity (per second) detected along the length of the capillaries, and shows automatically detected peaks, that can be quantified by calculation of the area under the curve (AUC). We have optimised Wes for dystrophin quantification in human skeletal muscle samples, making use of a healthy control sample with average dystrophin levels as a reference control and α-actinin as a protein loading/muscle content control. We demonstrate here that dystrophin quantification by Wes is highly sensitive, reproducible and quantitative over a large dynamic range and have applied it to quantify dystrophin expression in skeletal muscle samples of a large panel of healthy controls and individuals with BMD and DMD.

## Materials and methods

### Human skeletal muscle samples

Prior to initiation and commencement of this study written informed consent for research use was obtained from all subjects or their legal representatives and all skeletal muscle samples were approved for research use by the local institutional research ethics review boards or medical ethics committees of the University Medical Centres, University Hospitals or Institutes listed below. Human control muscle samples were obtained from a commercial tissue bank (Asterand, Hertfordshire, UK; http://asterand.com/Asterand/about/ethics.htm; *control19*), Myobank AFM (Institute de Myology, France; control5), Vrije Universiteit Medical Center (VUMC, Amsterdam, the Netherlands; *control0*, *6–9 and 16–18*) and Leiden University Medical Center (LUMC, Leiden, the Netherlands; *control1-4*, *10–15*, *20–31*). All BMD biopsies were obtained from Leiden University Medical Center (LUMC, Leiden, the Netherlands; *BMD1-25*). DMD biopsies were obtained from patients participating in BioMarin clinical studies (NCT02958202 (BMN044); NCT01826474 (BMN045); NCT01957059 (BMN053); *DMD1-12*), University Hospital of Leuven (*DMD15-17*) and Myobank AFM (Institute de Myology, France; *DMD13-14*).

A full overview of the skeletal muscle samples used in this study (mostly derived from tibialis, gastrocnemius, quadriceps or biceps) is given in S1 Table.

### Protein lysate preparation from muscle biopsies

Skeletal muscle biopsies were frozen in 2-methylbutane cooled in liquid nitrogen (clinical samples) or were frozen directly in liquid nitrogen and stored at -80°C or in liquid nitrogen until further use. Protein lysates from muscle tissue were prepared by sectioning or cutting off small pieces of in total ~ 5–10 μg, placing them in a Magnalyser vial without beads (MagNA Lyser Green Beads; Roche 03358941001) and adding 100 μl Protein Lysis Buffer (15% SDS, 75 mM Tris-HCl pH 6.8, 1 Protease Inhibitor Cocktail tablet (Roche/Sigma 04693159001)/8 ml; 5% β-Mercaptoethanol). After briefly spinning down, approximately 15 beads were added to each tube. The samples were then homogenized by 2–4 cycles (20 seconds; 7000 rpm) in the MagNA Lyser Instrument (Roche 03358976001) and spun down for 5 min at 13000 rpm. The supernatant was supplemented with glycerol (final concentration 20%) and then samples were stored at -80°C until further use.

To measure total protein concentration, 20x dilutions of the lysates were precipitated using the Compat-Able Protein Assay Preparation Reagent kit (Thermo Scientific 23215) to eliminate interfering substances from protein assay samples and make them compatible with the BCA assay. Concentrations were measured using the Pierce BCA Protein assay kit (Thermo Scientific 23227), according to the manufacturer’s instructions.

### Wes procedure

Wes analysis was performed on a Wes system (ProteinSimple, product number 004–600) according to the manufacturer’s instructions using a 66–440 kDa Separation Module (ProteinSimple SM-W006 or SM-W008) and either the Anti-Rabbit Detection Module (ProteinSimple DM-001) or the Anti-Mouse Detection Module (ProteinSimple DM-002), depending on the primary antibody used. In brief, skeletal muscle samples were diluted to an appropriate concentration (25 μg/ml for healthy control and BMD; 250 μg/ml for DMD, which results in loading amounts of 0.125 and 1.25 μg respectively) in sample buffer (100x diluted ‘10x Sample Buffer 2’ from the Separation Module), then mixed with Fluorescent Master Mix and heated at 95°C for 5 min. The samples, blocking reagent (antibody diluent), primary antibodies (in antibody diluent), HRP-conjugated secondary antibodies and chemiluminescent substrate were pipetted into the plate (part of Separation Module). Instrument default settings were used: stacking and separation at 475 V for 30 min; blocking reagent for 5 min, primary and secondary antibody both for 30 min; Luminol/peroxide chemiluminescence detection for ~15 min (exposures of 1-2-4-8-16-32-64-128-512s). The resulting electropherograms were inspected to check whether automatic peak detection required any manual correction. Identification of specific peaks becomes more difficult when dystrophin levels are close to background. In particular in DMD samples the background is not only higher due to loading of 10-fold more protein, but also due to the presence of various dystrophin isoforms, and revertant and trace dystrophin with variable size. The following criteria were used to discriminate low dystrophin signals from background: The peak signal-to-noise (S/N) ratio given by the software must be ≥10, and the peak height/baseline ratio (calculated manually from the peak height and baseline values given by the software) must be ≥3.

To control for differences in signal between experiments, a 6-point calibration curve of a healthy muscle reference sample was routinely included, usually ranging from 0.008–0.250 μg or 0.004–0.125 μg, depending on the samples analysed. This reference sample was selected from a panel of healthy controls based on it displaying average dystrophin levels with both ab154168 and Mandys106. The following criteria were applied to each calibration curve: the 0.125 μg point must have a chemiluminescence peak of >150.000 arbitrary units (to be determined for each Wes system), and the linearity of the curve must have an R^2^ > 0.99, based on 4–6 points in a relevant concentration range.

In addition, to control for muscle content, the sample dilutions used for dystrophin detection were further diluted to a concentration of 2.5 μg/ml total protein (0.0125 μg loaded) and were subsequently analysed for α-actinin levels. For this α-actinin analysis a calibration curve was also included, ranging from 0.0008–0.025 μg of the same healthy control as used for dystrophin analysis. The final muscle content-corrected dystrophin values were generated by first expressing both the dystrophin and α-actinin signals as percentage of control (% CTRL; using a calibration curve) using the following formula:

$$\%CTRL=sample\;protein\;equal\;to\;x\frac{\mu g}{ml}CTRL(from\;cal.curve)÷x\frac{\mu g}{ml}sample\;loaded$$

and then dividing the dystrophin % CTRL value by the α-actinin % CTRL value.

**Fig 1:**
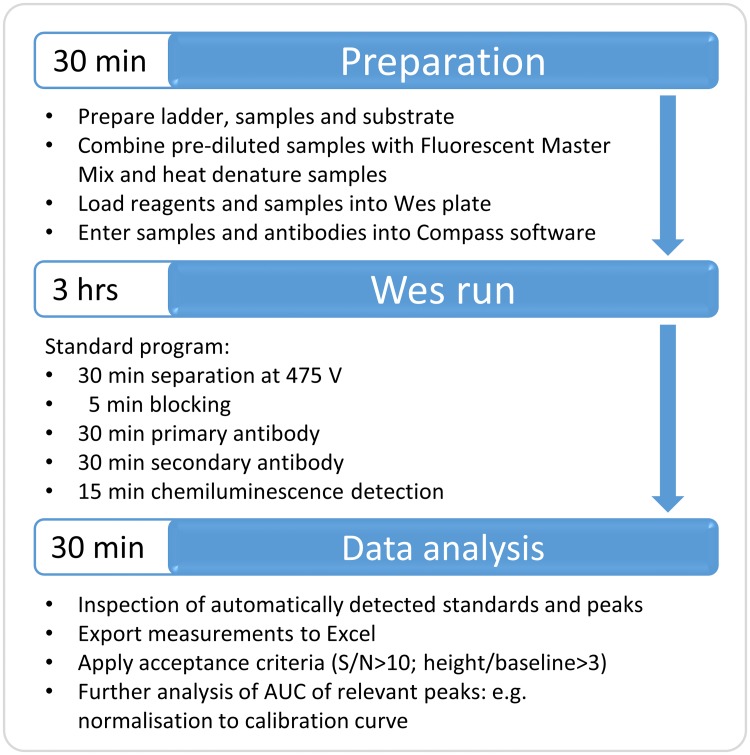
Workflow of Wes procedure. The required steps and time to complete a Wes run and analysis are indicated.

An overview of the workflow for Wes is depicted in Fig 1.

### Antibodies

Four different anti-dystrophin antibodies were tested for their signal in Wes, recognising different epitopes (Table 1). Antibodies Mandys106 and Manex59B were kindly provided by Dr. Glenn Morris. In addition, an antibody targeting α-actinin was used to control for loading and muscle content of the loaded sample. Optimal dilutions of each antibody were determined and are described in the Results section and listed in S2 Table.

**Table 1:** Overview of dystrophin antibodies.

Antibody	Reference	Host	Dystrophin specificity	Dystrophin epitope	Secondary antibody
Anti-dystrophin	Abcam ab154168	rabbit monoclonal	human and mouse	C-terminus	Anti-rabbit-HRP (Wes detection kit)
Mandys106	Licensed from Glenn Morris	mouse monoclonal	human-specific	Exon 43	Anti-mouse-HRP (Wes detection kit)
Manex59B	Licensed from Glenn Morris	mouse monoclonal	human and mouse	Exon 59	Anti-mouse-HRP (Wes detection kit)
NCL-DYS-1	Leica DYS1-CE	mouse monoclonal	human and mouse	Exon 26–29	Anti-mouse-HRP (Wes detection kit)

### Western blot

Samples including a healthy muscle reference sample were diluted to an appropriate concentration with milli-Q, then 4x Laemli Sample Buffer (Biorad) was added (ratio 3:1) and the samples were denatured at 95°C for 5 min. SDS-PAGE was performed in a cold room (4°C). The samples were loaded onto a 3–8% gradient Tris acetate poly-acrylamide gel (Bio-rad Criterion XT) and run for 4 hours at 100V (~50 mA) in Invitrogen 20x Novex Tris-Acetate SDS Running Buffer. The proteins were then blotted on to a 0.2M nitrocellulose membrane, using a Turbo Transfer pack in combination with the Trans Blot Turbo Transfer System (Bio-rad), for 15 min at 25V. The following incubations were performed, with TBST wash-steps in between: 1h blocking at RT (TBS-5% milk powder)—overnight primary antibody incubation in cold room (1/2000 for ab154168 in TBST and 1/125 for Mandys106 in TBST)– 15 min blocking at RT– 1h secondary antibody incubation at RT (1/5000 anti-rabbit-IRDye 800CW for ab154168 and 1/5000 anti-mouse-IRDye 800CW for Mandys106 in TBST). Membranes were scanned on the Odyssey system (Li-COR).

To control for loading and muscle content, the blots were re-probed for α-actinin. After dystrophin detection, the membranes were incubated overnight in a cold room with an anti-α-actinin antibody (ab68167, Abcam) at a 1/3000 dilution, followed by 1hr at RT with secondary antibody anti-rabbit-IRDye 800CW (Li-Cor 925–32211) at a 1/5000 dilution. Protein bands were visualized with Odyssey. As this was a mostly qualitative experiment, no calculations were made (muscle content comparability was judged by visual inspection of the α-actinin signal).

## Results

### Bridging from Western blot to Wes analysis

**Fig 2:**
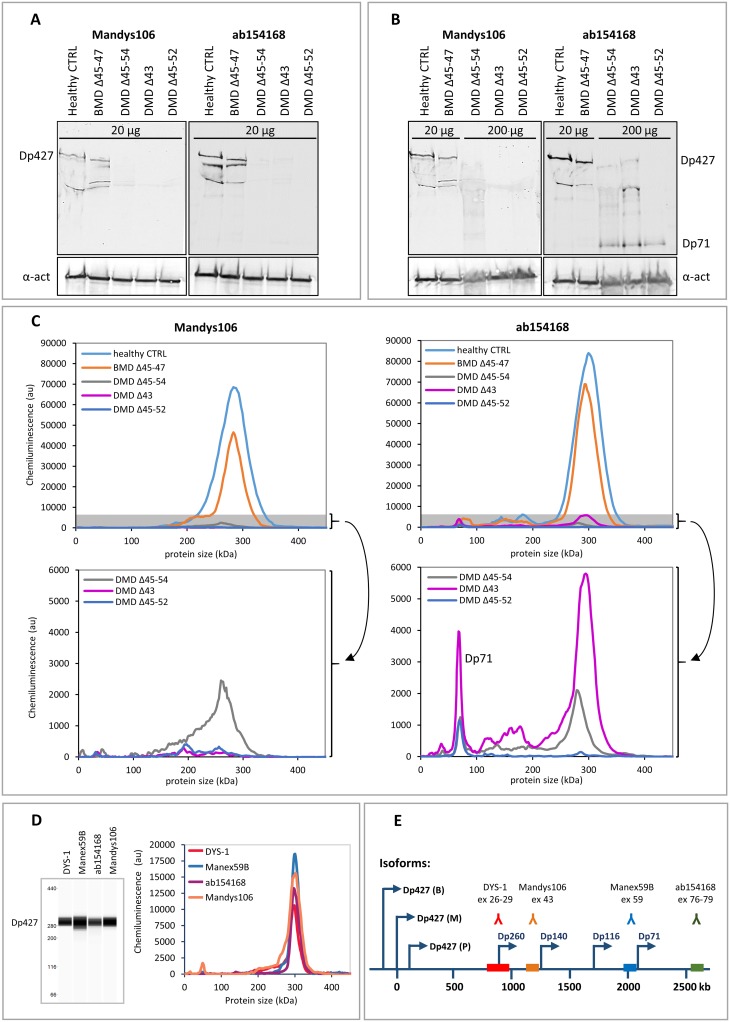
Comparison of dystrophin detection by Western blot and Wes in skeletal muscle samples from a healthy control, a BMD patient and 3 DMD patients. Western blot analysis of a healthy control, a BMD and 3 DMD samples. Expected molecular weights: BMD del45-47: 410 kDa, DMD del45-54: 367 kDa, DMD del43: 422 kDa, DMD del45-52: 381 kDa. A) A dystrophin signal is visible of the expected size (427 kDa) in the healthy control and BMD samples when loading 20 μg per lane, but not for the DMD samples. B) Loading of 10x more material (200 μg per lane) results in a weak dystrophin signal for 2 out of the 3 DMD samples using ab154168 and no signal using Mandys106. The lower panel shows the α-actinin signal. Note that the middle DMD sample has a deletion of exon 43 (epitope for Mandys106), so it is not expected to produce a signal. The additional bands of lower molecular weight are either degradation products and/or dystrophin isoforms. C) Wes analysis of the same samples: At 1 μg total protein (5 μl of a 200 μg/ml dilution) clear signals are detected at 300 kDa for all but the lowest DMD sample using ab154168. The bottom graphs show the DMD data again, only with adjusted y-axes, to zoom in on the grey shaded areas of the upper graphs. D) Virtual blot and electropherogram representation of a typical full length dystrophin signal in a healthy control sample detected by Wes. The dystrophin signal is detected using 4 different antibodies: Dys-1 (1/25 antibody, loaded 0.04 μg protein), Manex59B (1/100 antibody, loaded 0.15 μg protein), ab154168 (1/1000 antibody, loaded 0.13 μg protein) and Mandys106 (1/50 antibody, loaded 0.13 μg protein). E) An overview of the dystrophin antibody epitopes recognised by the different antibodies and the exons coding for the epitopes. The arrows indicate the promotors of the different dystrophin isoforms (Dp427: brain (B), muscle (M) and Purkinje (P); Dp260, Dp140, Dp116 and Dp71).

To bridge from traditional WB analysis to the more state of the art Wes analysis, skeletal muscle samples from a healthy control, a BMD patient and 3 DMD patients with different mutations were analysed using both methods. Using WB the full length dystrophin band was visible in the healthy control at the expected size of 427 kDa for antibodies Mandys106 and ab154168 (Fig 2A and 2B). The dystrophin signal in the BMD sample was also visible with both antibodies, but was slightly shorter than 427 kDa, as expected, due to the deletion of exons 45 to 47 (Fig 2A and 2B). No bands were visible for 3 different DMD samples when the same amount of total protein (20 μg) was loaded (Fig 2A), but loading 10x more sample (200 μg) resulted in weak intensity bands for 2 of the 3 DMD samples with ab154168 (Fig 2B). Using Mandys106 no signal was visible in any of the 3 DMD samples. It must be noted, however, that the DMD sample with the deletion of exon 43 lacks the epitope detected by Mandys106, so lack of signal for this DMD sample was expected, despite the loading of 10x more sample. The 200 μg of DMD sample required to give a dystrophin signal on WB likely affects the migration of the samples in the gel. In addition, the large size of dystrophin makes it notoriously difficult to transfer efficiently onto membrane during blotting. Consequently, the bands detected for the DMD samples on WB are vague, irregular and reproducibility is poor, making the procedure not very quantitative.

Next the same samples were subjected to Wes analysis. Wes analysis was much more sensitive, requiring only a fraction of the amount of sample to give a dystrophin signal. A sharply defined chemiluminescent dystrophin signal was quantifiable by calculation of the area under the curve (AUC) in the healthy control and BMD sample, and in 2 of the 3 DMD samples using ab154168 and only ~1 μg of total protein (Fig 2C), which is 200x less sample material than was used for WB. While the chemiluminescent signals were low for the DMD samples, as can be expected, the signal-to-noise (S/N) ratios were well above the proposed threshold value of 10 for all DMD samples, ranging from 51–507 for ab154168 and from 33–71 for Mandys106. No dystrophin signal was detected using Mandys106 in the DMD sample with the exon 43 deletion.

The dystrophin signal detected by Wes displayed a molecular weight of approximately 300 kDa, which is lower than the predicted 427 kDa for the full-length (muscle) dystrophin isoform. This size discrepancy can be explained by the molecular weight ladder used in Wes, which underestimates molecular weights above 280 kDa (S1 Fig). The specificity of dystrophin detection by Wes was however confirmed by the fact that 4 different antibodies against dystrophin which recognise different dystrophin epitopes, Mandys106, ab154168, Manex59B and Dys1, all resulted in a signal at 300 kDa in a healthy control sample (Fig 2D).

Wes analysis also allowed for identification of dystrophin isoforms, including the Dp71 isoform which was clearly visible and quantifiable in all 3 of the DMD samples using ab154168 (Fig 2C). Using Mandys106 the Dp71 isoform was not detected, because this isoform has its promotor in exon 63 [25] and therefore lacks the Mandys106 epitope (exon 43; Fig 2E).

In conclusion, Wes analysis is more sensitive than WB and allows for dystrophin quantification in 1 μg of loaded protein from skeletal muscle samples derived from healthy controls, BMD and DMD patients, which is 20-200x less material than required for WB. Obtained chemiluminescent signals are well above background and easily quantifiable by calculation of the AUC.

### Optimization of total protein and antibody concentrations for Wes

**Fig 3:**
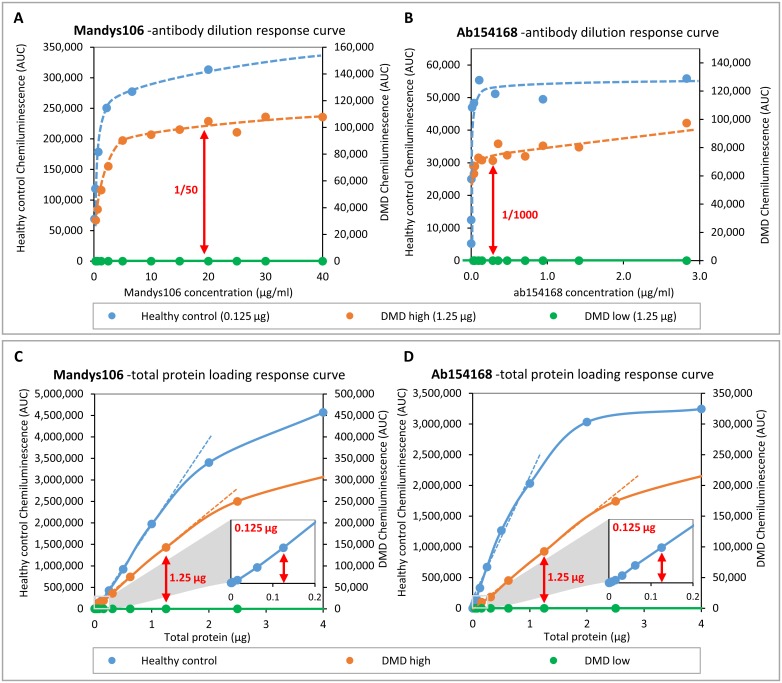
Optimization of antibody dilution for Mandys106 and ab154168 and determining linearity of quantification. Skeletal muscle samples from a healthy control and a DMD patient with high trace dystrophin and a DMD patient with low or complete absence of dystrophin were used for optimisation and to determine the linear range of quantification. A and B) Dystrophin detected in healthy control (25 μg/ml) and DMD (250 μg/ml) samples using different antibody dilutions. C and D) Dystrophin detected in different amounts of healthy muscle lysate, using the antibody concentrations established in -Aand B (Mandys106: 1/50; ab154168: 1/1000). The small squares at the bottom right of graphs C and D represent a zoomed-in section of the low protein concentration range, to show signal linearity between 0 and 0.2 μg total protein. The arrows indicate the dilutions/concentrations selected for further experiments.

To set up Wes as a new standard for dystrophin quantification, we optimized and validated: 1) the optimal antibody dilution to be used to achieve a maximum dystrophin signal at a fixed amount of protein loaded and 2) the range of protein to be loaded at the optimal antibody dilution to ensure linearity of the dystrophin signal. Two antibodies were selected: rabbit monoclonal ab154168 because of its C-terminal epitope and strong signal with very little background, and Mandys106 because of its epitope in the central rod domain and specificity for human dystrophin. The antibodies were titrated using a healthy control sample and 2 different DMD samples with either low or (relatively) high dystrophin levels. The optimal antibody concentration was considered to be a saturating dilution in which addition of more antibody would not further increase the signal, implying coverage of all free epitopes. For Mandys106 a 1/50 dilution was required while for ab154168 an antibody dilution of 1/1000 was enough to reach a dystrophin signal plateau (Fig 3A and 3B). This was observed when loading 0.125 μg of a healthy control sample as well as 1.25 μg of a DMD sample with a relatively high trace dystrophin level (DMD high; exon 45 deletion). No signal was observed for the second DMD sample (DMD low; exon 48–52 deletion), even at high antibody concentrations, indicating that it has very low or complete absence of dystrophin.

Next, the optimal amount of protein to be loaded was determined, using the selected antibody dilutions. In order to define the linear range of detection, a total protein concentration dilution series of the same healthy control and DMD samples was loaded. This experiment showed that Wes is capable of detecting dystrophin at very low concentrations. For the healthy control, chemiluminescence increased linearly with protein concentration from 0.002 μg up to 1.25 μg for Mandys106 (Fig 3C) and from 0.0005 μg up to 1 μg for ab154168 (Fig 3D). Above that, the chemiluminescence started to level off, implying signal saturation. Loading of 0.125 μg was chosen as the optimal amount of protein to be loaded of healthy control skeletal muscle samples for the next experiments. For the high DMD sample the linearity ranged from 0.04–1.8 μg for Mandys106 (Fig 3C) and for ab154168 from 0.16 μg to 2.5 μg (Fig 3D). Above this the signal leveled off, while the chemiluminescence was not even 10% of that of the healthy control, suggesting capillary saturation rather than substrate depletion as the cause. Loading of 1.25 μg, so 10-fold more than of healthy controls, was chosen as the optimal amount of protein to be loaded of DMD skeletal muscle samples for the next experiments to maximize the ability to detect a dystrophin signal by loading as much protein as possible without saturating the capillaries. No dystrophin at all was detectable even after loading 5 μg of protein for the low DMD sample for both the Mandys106 and ab154168 antibodies.

### Linear range of Wes for dystrophin quantification in DMD samples

**Fig 4:**
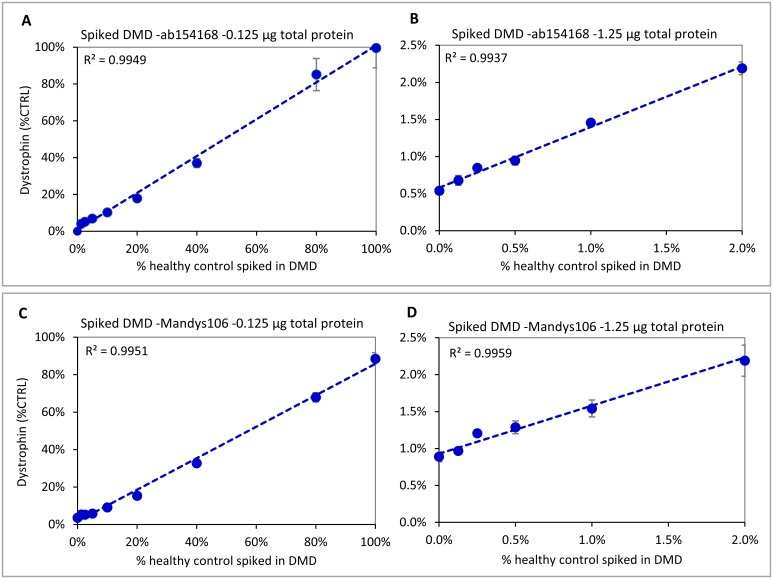
Dynamic ranges for ab154168 and Mandys106 using different amounts of healthy control sample spiked into a DMD sample. Both antibodies show good linearity from ~5%-100% of healthy control when loading 0.125 μg (A and C). When loading 1.25 μg (B and D), ab154168 shows good linearity down to 0.125% of healthy control, while for Mandys106 samples with dystrophin levels ≤0.5% of control become hard to distinguish, due to the higher aspecific background signal. Experiments were performed in triplicate.

Since the amount of protein to be loaded for DMD samples analysis is relatively high for Wes (1.25 vs 0.125 μg for healthy control), we investigated whether this may cause aspecific background signal or interfere with sample running efficiency in the capillary. To test the limits of detection while keeping the total protein amount loaded constant, different ratios of a healthy control skeletal muscle protein lysate were spiked into lysate from a DMD patient with low trace dystrophin. When loading 0.125 μg total protein, dystrophin amounts ranging from 1.25% -100% of healthy control could be linearly detected using ab154168 (so the upper limit of detection, or ULOD, was 100%) (Fig 4A). To see the effect of loading 10-fold more protein and determine the lower limit of detection (LLOD), 1.25 μg of the same DMD sample was spiked with a range of 0–2% of healthy control. This resulted in a good linear range of detection using ab154168 (Fig 4B). The DMD sample used for spiking contained some trace dystrophin: This ‘0% spiked’ sample thus showed a dystrophin level of about 0.5% of CTRL with a coefficient of variation (CV; standard deviation/average) between 3 experiments of 15%, which means that the LLOD and lower limit of quantification (LLOQ) are equal or lower than 0.5% of CTRL. Differences between samples spiked with 0%, 0.125% and 0.25% of healthy control were distinguishable and the detected levels were accurate after deduction of the 0.5% trace dystrophin contributed by the DMD sample (Table 2). An additional experiment using 1.25 μg of loaded protein, allowed the linear quantification of spiked samples up to 100% healthy control, indicating a wide dynamic range (S2 Fig).

**Table 2:** Linearity of dystrophin quantification in a DMD sample spiked with different amounts of healthy control sample.

Sample loading		Ab154168			Mandys106		
total protein	% control spiked	dystrophin (% CTRL)	S/N	Dystrophin detection range	dystrophin (% CTRL)	S/N	Dystrophin detection range
**0.125μg**	100%	100%	1166	4%–100%	88%	432	3.6%–88%
	80%	85%	1271		68%	385	
	40%	37%	630		33%	213	
	20%	18%	309		15%	67	
	10%	10%	111		9%	37	
	5%	6.9%	64		5.8%	17	
	2.5%	5.1%	43		5.2%	13	
	1.25%	4.1%	16		5.3%	17	
	0%	BLQ	NA		3.6%	12	
**1.25 μg**	4%	NT	NT	0.5%–2.2%	3.6%	153	0.9%–3.6%
	2%	2.2%	232		2.2%	55	
	1%	1.5%	124		1.5%	32	
	0.5%	0.95%	90		1.3%	17	
	0.25%	0.85%	52		1.2%	13	
	0.125%	0.68%	58		1.0%	8	
	0%	0.54%	28		0.9%	11	

Linear detection ranges for healthy control spiked into a DMD sample, expressed as % of CTRL, for ab154168 and Mandys106. At 250 ug/ml, the highest sample tested was 2% spiked. NT: not tested. S/N: signal to noise ratio. BLQ: below limit of quantification.

Similar experiments with Mandys106 showed more aspecific background staining, leading to less sensitivity at low dystrophin concentrations. Loading of 0.125 μg gave a linear response for DMD samples spiked with 5%-100% of healthy control (Fig 4C). Below that the samples still gave a signal, but were difficult to distinguish from each other. When loading 1.25 μg, a linear response was found for DMD samples spiked with 0%-2% of healthy control (Fig 4D), as well as for samples spiked with 0%-100% of healthy control (S2 Fig). Although the S/N ratio of some measurements for the 0%-0.25% samples was close to or below 10, the CV between experiments was still <25%. The dystrophin level measured for the DMD sample (not spiked with healthy control) using Mandys106 was 0.9%, so higher than with the more specific ab154169 where this was 0.5% (Fig 4D and Table 2).

### Reproducibility of Wes

To determine the reproducibility (precision) of the method, an experiment was set up to address several sources of variability. An important source of intra-assay variation is the pipetting (dilution) error, while inter-assay variation can be caused by inter-operator variation, differences between antibody batches, small differences in Wes performance (sample running and signal detection) and differences between different Wes systems. To test this, 3 different dilutions of the healthy control were made and aliquoted (2.5 μg/ml, 12.5 μg/ml, and 37.5 μg/ml), and designated low, medium or high based on the amount of dystrophin. Three independent dilutions of the low, medium and high samples were prepared per experiment and quantified in duplicate by 2 different operators for both antibodies. These experiments showed that the reproducibility of the assay is high but may be influenced by the (primary) antibody used. The raw chemiluminescence values were higher for Mandys106 than for ab154168, especially for the low sample measurements. The reproducibility was better for ab154168 than for Mandys106 (Table 3). For ab154168 the CV between chemiluminescence signals from duplicate wells was mostly below 10%, with a few outliers of up to 24% (Table 3). After conversion to μg/ml CTRL using the control calibration curve, the intra-assay CV was determined by the CV between dilutions for each sample: for ab154168 the dilution CV ranged from 0%-11%. The inter-assay CV per operator was 4%-12%, while the inter-operator variation was 4–19%. The overall assay variation, calculated over all 18 measurements for each sample (3 dilutions x 3 experiments x 2 operators) was 8%-16%.

**Table 3:** Assay variation using ab154168 and Mandys106.

**ab154168**		**intra-assay variation**						**inter-assay variation (1 operator)**						**inter-operator & overall assay variation**					
		**L**		**M**		**H**		**L (3 exp)**		**M (3 exp)**		**H (3 exp)**		**L (2 operators)**		**M (2 operators)**		**H (2 operators)**	
		**(n = 3 dilutions)**		**(n = 3 dilutions)**		**(n = 3 dilutions)**													
		mean	CV	mean	CV	mean	CV	mean	CV	mean	CV	mean	CV	mean	CV	mean	CV	mean	CV
Operator 1	plate 1	1.9	2%	9.8	4%	30.1	3%	1.9	4%	10.3	6%	32.5	6%	2.0	4%	9.1	19%	31.2	6%
	plate 2	2.0	1%	11.1	2%	34.6	6%												
	plate 3	1.8	0%	10.1	2%	32.8	3%		(5%)		(7%)		(8%)						
	duplicates	6% (0.5%-16%)		5% (0.2%-10%)		5% (0.04%-17%)													
Operator 2	plate 1	2.0	11%	7.1	4%	29.7	6%	2.1	12%	7.9	11%	29.9	4%		(9%)		(16%)		(8%)
	plate 2	1.8	2%	7.7	5%	28.7	9%												
	plate 3	2.3	2%	8.9	7%	31.3	6%		(12%)		(11%)		(7%)						
	duplicates	3% (0.2%-7%)		8% (2–24%)		9% (4–19%)													
**Mandys106**		**intra-assay variation**						**inter-assay variation (1 operator)**						**inter-operator & overall assay variation**					
		**L**		**M**		**H**		**L (3 exp)**		**M (3 exp)**		**H (3 exp)**		**L (2 operators)**		**M (2 operators)**		**H (2 operators)**	
		**(n = 3 dilutions)**		**(n = 3 dilutions)**		**(n = 3 dilutions)**													
		mean	CV	mean	CV	mean	CV	mean	CV	mean	CV	mean	CV	mean	CV	mean	CV	mean	CV
Operator 1	plate 1	2.3	6%	12.9	7%	41.5	11%	2.2	6%	13.3	2%	43.3	3%	2.6	26%	11.7	20%	37.7	21%
	plate 2	2.0	16%	13.5	7%	44.5	5%												
	plate 3	2.1	24%	13.5	3%	43.9	2%		(11%)		(6%)		(7%)						
	duplicates	15% (6–32%)		7% (2%-19%)		6% (0.5%-13%)													
Operator 2	plate 1	3.2	1%	9.7	4%	32.5	3%	3.1	3%	10.1	4%	32.1	3%		(21%)		(15%)		(16%)
	plate 2	3.0	3%	10.5	4%	32.7	6%												
	plate 3	3.1	4%	10.0	2%	31.1	3%		(4%)		(5%)		(4%)						
	duplicates	3% (0.4%–5%)		7% (0.1%–24%)		13% (2%–22%)													

Dystrophin levels quantified in 3 different dilutions of a healthy control skeletal muscle sample, designated L (low; 2.5 μg/ml), M (medium; 12.5 μg/ml) and H (high; 37.5 μg/ml) expressed as μg/ml CTRL. For inter-assay and inter-operator variation 2 different CVs are given: in the inter-assay variation column, the upper CV is between the averages for the whole experiments (3 values), while the CV between brackets is for all separate dilutions within those experiments (3x3 = 9 values). In the inter-operator variation column, the upper CV is between the averages for the 2 operators (2 values), while the CV between brackets is between all separate dilutions within those experiments (2x9 = 18 values); this is also referred to as ‘overall CV’. For ab154168 the accuracy (% of healthy control amount loaded) was 80%, 73% and 83% for the L, M and H sample. For Mandys106 this was 104%, 94% and 101%, respectively.

For Mandys106 the average duplicate well CV based on chemiluminescence was 3%-15%, with outliers of up to 32% (Table 3). After conversion to μg/ml CTRL, the intra-assay CV ranged from 1%-24%. The inter-assay CV per operator was 2%-6%, while the inter-operator variation was 20%-26%. The overall assay variation was 15%-21%.

### Normalizing for loading and muscle content: α-actinin

For dystrophin detection by Western blot, a muscle-specific protein unaffected by DMD is often used to correct for differences in sample loading or in muscle content of the samples. Alpha-actinin is a cytoskeletal actin-binding protein, localizing to the Z-disks in striated, cardiac and smooth muscle cells, where it stabilizes the muscle contractile apparatus [26]. It is not part of the DGC complex and does not appear to be affected by DMD disease progression [27], making it a suitable protein to control for differences in loading or in muscle content caused by fibrotic and/or adipose regions within skeletal muscle samples.

To allow use of α-actinin as a normalisation protein in Wes, α-actinin antibody ab68167 was optimized similarly to the dystrophin antibodies described above (S3 Fig and S2 Table). Since expression of α-actinin is much higher than dystrophin, samples required an extra dilution step of 10-fold for healthy control muscle, or 100-fold for DMD muscle. To facilitate comparison of data between experiments, an α-actinin calibration curve was routinely included, usually ranging from 0.025 to 0.0008 μg. Normalizing to α-actinin did not affect the reproducibility of the dystrophin measurements (data not shown).

### Analysis of human healthy control, BMD and DMD samples

**Fig 5:**
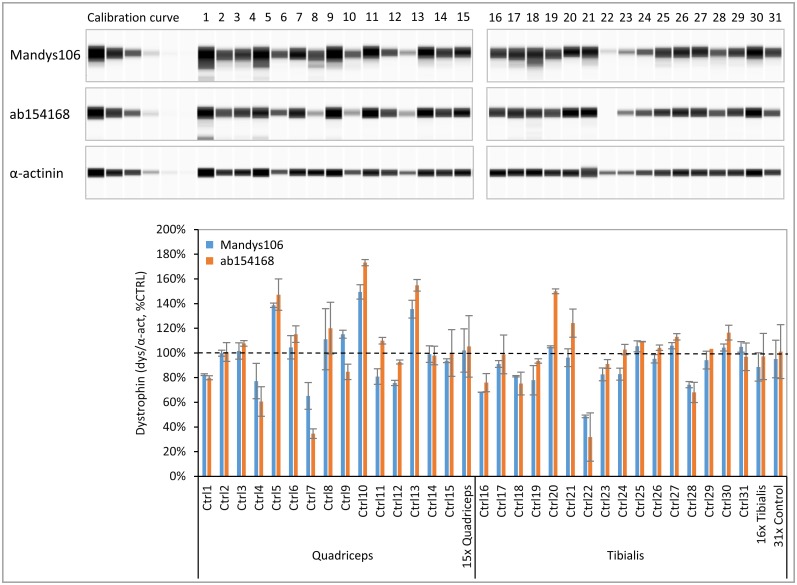
Dystrophin levels in skeletal muscle samples derived from healthy controls. A panel of 31 healthy human tibialis and quadriceps muscle samples was analysed for dystrophin using ab154168 and Mandys106 and normalized to α-actinin (n = 2). Protein loading was 0.125 μg. A) Virtual blot view of Wes result (lanes compiled from 2 runs) showing the dystrophin levels obtained with antibodies Mandys106 and ab154168 (top panels) and the α-actinin signal (lower panel). B) Dystrophin levels (divided by α-actinin) expressed as a percentage of the control (% CTRL). In addition to the individual sample values, the average dystrophin levels of all 15 quadriceps samples, the 16 tibialis samples and all 31 samples are displayed. The dashed line represents 100% of CTRL.

To use dystrophin as a biomarker in drug development programs, a standard healthy control sample with a representative (average) dystrophin expression is required to define 100% levels. In a pilot experiment on 14 healthy human muscle biopsies we selected a sample that had average control dystrophin levels and of which sufficient material was available for numerous Wes analyses. Using our optimized Wes protocol we determined the range of dystrophin expression in an extended panel of 31 healthy human muscle biopsies. These biopsies were mostly derived from quadriceps or tibialis muscle and were obtained from both male and female donors ranging from age 22 to 75 years (Fig 5). Dystrophin levels normalized for α-actinin were expressed relative to the selected control (% of CTRL) and ranged from 49% to 149% for Mandys106, which is a 3-fold difference between the highest and the lowest control. For ab154168 the dystrophin levels ranged from 32% to 173%, which is a 5-fold difference between the highest and the lowest control sample. The average, as well as the median of all 31 healthy controls was 95% for Mandys106 and 101% for ab154168, indicating a non-skewed distribution, and confirming that the selected control (set to 100%) indeed has average dystrophin levels. This control sample was thus used as standard control sample (CTRL) in all subsequent experiments. No difference in dystrophin levels was observed between male and female donors nor between biopsies from quadriceps and tibialis muscles. Furthermore, no correlation was observed between dystrophin expression and age, although it should be noted that this sample set was relatively small and did not include any skeletal muscle samples derived from children.

**Fig 6:**
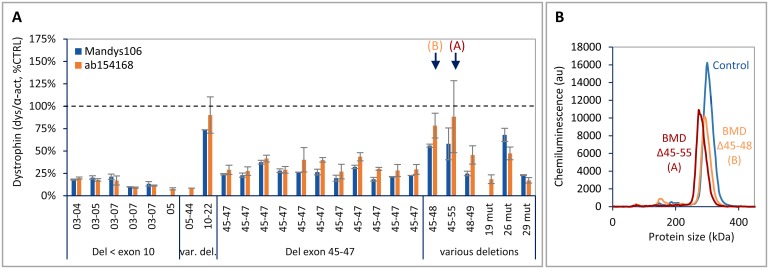
Dystrophin levels in skeletal muscle samples derived from BMD patients. Panel of 25 tibialis samples from BMD patients, analysed for dystrophin using ab154168 and Mandys106 and normalized to α-actinin (n = 2). Protein loading was 0.125 μg. A) Dystrophin levels (divided by α-actinin) expressed as a percentage of healthy control (% CTRL). The 2 BMD samples indicated by an arrow are depicted in B. B) Electropherogram showing the molecular weight shift of the peaks for mutant dystrophin with different sizes derived from the 2 BMD samples (A and B) indicated by an arrow in A, compared to a control sample with full-length dystrophin. BMD A (del 45–55: calculated MW = 358 kDa, expected apparent MW on Wes is 279 kDa; BMD B (del 45–48): calculated MW = 401 kDa, expected apparent MW on Wes is 294 kDa.

Dystrophin levels were also determined in a panel of 25 BMD tibialis muscle samples with different dystrophin mutations. Dystrophin levels ranged from 10% to 90% of CTRL, with individuals with a deletion in the actin binding domain (exons 2–8) displaying the lowest dystrophin levels (Fig 6A). Although the results were similar for both dystrophin antibodies, in most samples dystrophin detection was slightly higher using ab154168. In addition to quantifying dystrophin levels, Wes analysis also allowed detection of size differences between mutant dystrophin proteins from BMD samples with different deletions (Fig 6B).

**Fig 7:**
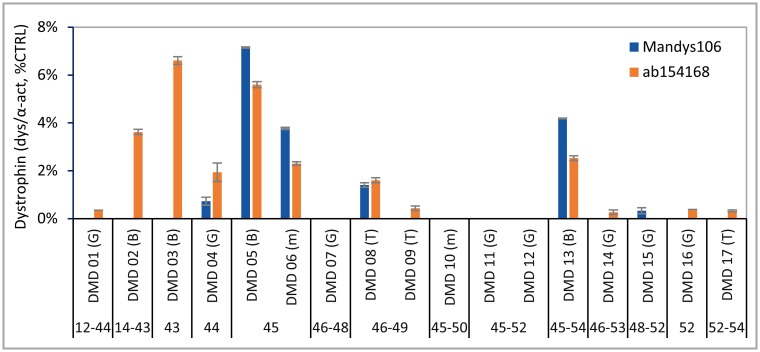
Dystrophin levels in skeletal muscle samples derived from DMD patients. Panel of 17 gastrocnemius, biceps and tibialis muscle samples from DMD patients, analysed for dystrophin using ab154168 and Mandys106 and normalized to α-actinin (n = 2). Protein loading was 1.25 μg. Muscle types are indicated between brackets: G = gastrocnemius; B = biceps; T = tibialis and M = miscellaneous.

Finally, a total of 17 DMD skeletal muscle samples were analysed by Wes (Fig 7). These included pre-treatment samples (gastrocnemius, biceps or tibialis) from boys aged 6.5–16 enrolled in 4 clinical trial studies (by BioMarin and the University of Leuven; see Materials and methods), with mutations amenable to antisense oligonucleotide-induced exon 44, exon 45 or exon 53 skipping. Trace dystrophin levels were detected with at least one of both antibodies in 13 out of the 17 samples and ranged from 0.2% to 7% of CTRL, depending on the antibody. Mutant dystrophin protein variants lacking exon 43 were not detected using Mandys106, while ab154168 did allow their identification and quantification, again demonstrating the specificity of Mandys106 for dystrophin and exon 43 (Fig 7, DMD samples 01–03). Although the number of DMD samples and distribution of mutations was limited, the highest dystrophin levels were found in patients with exon 44 flanking deletions (Fig 7, DMD samples 02–03, 05–06 and 13). The lowest dystrophin level detected (LLOD) was 0.4% of CTRL with ab154168 or 0.6% of CTRL with Mandys106.

**Fig 8:**
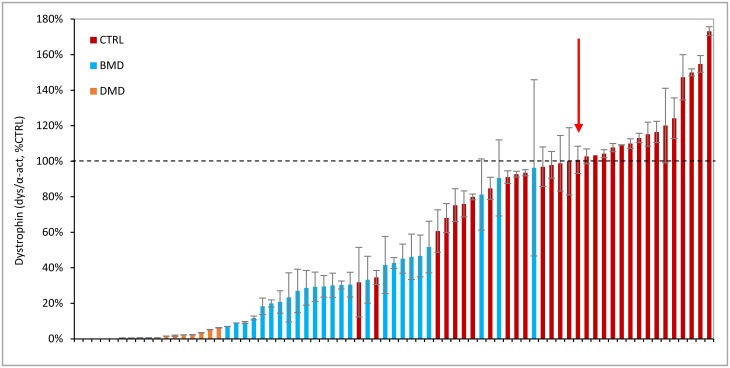
Summary of dystrophin levels in all healthy control, BMD and DMD samples analysed by Wes. Dystrophin levels obtained in all 31 healthy controls, 25 BMD and 17 DMD skeletal muscle samples using ab154168. The samples are normalized to α-actinin, expressed as a percentage of the standard control (% CTRL) and ranked from low to high dystrophin levels. DMD samples in orange, BMD in blue and healthy controls in red. The dashed line represents 100% CTRL; the red arrow shows the median of the healthy control samples, with a value of 101%.

The Wes-quantified levels of dystrophin in healthy controls, BMD and DMD samples were subsequently ranked from low to high (Fig 8). While there was a clear distinction between samples from DMD patients and healthy controls, there was some overlap between high BMD and low healthy control samples. Furthermore, there was only a small difference between dystrophin levels between the highest DMD and lowest BMD sample.

## Discussion and conclusion

To support the use of dystrophin as biomarker in clinical studies on investigational drugs aiming at dystrophin restoration in DMD patients, we have developed a quantitative method based on the ProteinSimple capillary immunoassay (Wes). The assay is highly sensitive and reproducible, linear over a wide dynamic range and with a LLOQ low enough to quantify trace dystrophin levels present in skeletal muscle of DMD patients.

The high assay sensitivity was demonstrated by LLOQs well below 1% of a healthy control, as well as the ability to detect differences in dystrophin levels as small as 0.125% of healthy control. The reproducibility for detecting dystrophin is in line with the supplier’s claim of a CV of 15% for intra-assay and 20% for inter-assay variation. While chemiluminescence results may vary between runs, the use of a calibration curve derived from a standard healthy control sample for quantification reduced overall variation to ≤16% (ab154168). This enables sensitive analysis of samples across almost the whole range of dystrophin levels from DMD (if above LLOQ) to healthy control within one experiment.

To control for differences in capillary loading and/or muscle content of samples in Wes, a housekeeping protein is essential, preferably one that has a constant expression in muscle cells regardless of disease status. Several potential candidate housekeeping proteins for muscle were selected for this purpose including α-actinin, vinculin and spectrin. For spectrin several antibodies were tested but none gave a specific signal on Wes. Compared to vinculin the α-actinin antibody gave the most clear signal with the strongest, cleanest peaks and, moreover, its expression appeared to be highly comparable between the BMD and DMD samples in this study, suggesting that its expression is not strongly affected by disease status. Therefore, we selected α-actinin for normalization. Both vinculin and α-actinin were expressed at too high levels to allow detection at the same protein concentration as dystrophin, requiring further dilution of the samples used for dystrophin detection, and therefore had to be run in separate capillaries. This is not preferred since it does not allow for correction for what exactly is loaded per capillary. However, any upstream error will be reflected in the α-actinin measurement, including errors in protein concentration measurement, calculation and pipetting errors. An improved approach that allows detection of both dystrophin and α-actinin at the same protein concentration (but not in the same capillary) is to dilute the secondary HRP-conjugated antibody used for α-actinin detection with unconjugated antibody (data not shown). An advantage of this is further reduction of variation introduced by pipetting errors during the dilution step (which would no longer be necessary).

The reproducibility of dystrophin quantification by Wes is in line with the FDA/EMA regulations for quantitative biochemical analysis: CVs between replicate measurements have to be below 20% or 25% (at LLOQ). For repeated/incurred sample analysis 2/3 of the results should be within 20% for small molecules and 30% for large molecules (calculated as ‘difference between results/average result’). Inter-assay variation per operator was well below 20% for both dystrophin antibodies tested. The inter-operator variation for Mandys106 was slightly higher (26% for the low dystrophin sample and 21% for the high dystrophin sample), which relates more to this specific antibody than to the Wes methodology itself.

So far Western blot (WB) in conjunction with immunofluorescence analysis (IFA) have been the most widely used methods to quantify dystrophin levels in biopsies from patients in clinical trials on dystrophin-restoring therapies. WB has a much smaller dynamic range for dystrophin quantification than Wes, since 10-fold more protein lysate of a (high) DMD sample needed to be loaded than of a healthy control muscle sample to allow detection of (trace) dystrophin. This is in contrast to Wes where the same amount of loaded protein was sufficient for dystrophin quantification, both in healthy control samples as well as in DMD samples (although we still suggest loading more for DMD samples, to improve sensitivity and reliability). The requirement for >100-fold less sample material is a big advantage of Wes, since patient biopsies can be very small (especially needle biopsies) and therefore limit the amount and type of analyses that can be performed. Quantification of dystrophin by Wes also has a much higher reproducibility compared to WB. In this study the overall assay variation, calculated over all 18 measurements for each sample (3 dilutions x 3 experiments x 2 operators) was 8%-16%, which is much lower than for WB: A multiple-laboratory study investigating the feasibility of using WB to measure dystrophin as a biomarker in DMD clinical trials showed a high assay variation, especially for low dystrophin expressing samples (81% inter-laboratory variation for a DMD sample; intra-laboratory CV up to 119%) [16]. Finally, Wes requires up to 500-fold less antibody per sample, provides a much faster workflow (hours rather than days for WB) and a more precise dystrophin quantification compared to WB.

We previously developed a semi-automated, objective and reproducible immunofluorescence analysis method optimized for assessing dystrophin levels per individual muscle fiber in an entire fiber population in muscle biopsies from BMD or DMD patients [17]. In this IFA method dystrophin intensity is measured in the sarcolemmal mask defined by spectrin for each individual muscle fiber, allowing only membrane-bound dystrophin to be taken into account. While IFA generally correlates well with WB for dystrophin levels ranging from DMD to BMD to healthy controls, IFA tends to overestimate dystrophin levels in DMD patients compared to healthy muscle [16,18,19]. Using Wes, no dystrophin could be detected in 4 out of 17 (23%) of the DMD samples tested, while using IFA we obtained a membrane signal in these 17 DMD samples (data not shown). While Wes is more quantitative than IFA, the latter ensures analysis of membrane-bound dystrophin only while easily allowing exclusion of revertant fibres and non-muscle tissue (fibrotic and fat tissue). For a comprehensive analysis of treatment effect, it is therefore recommended to perform both assays, with Wes analysis for quantitative results and IFA to confirm these results at the sarcolemma level.

DMD patients were historically diagnosed by absence of dystrophin as determined by qualitative assessment of muscle sections using immunofluorescence (IFA) [28]. However, more recent publications have reported low levels of trace dystrophin in most DMD patients, as well as the presence of dystrophin-positive revertant fibers [17,29]. Indeed, in this study using Wes we were able to detect dystrophin levels ranging from ~0.2% to 7% of CTRL in 13 out of 17 (76%) DMD samples tested, with only 4 DMD samples with no dystrophin at all detectable. The nature of the mutation can affect trace dystrophin levels and as we have previously reported we observed highest dystrophin levels in patients with exon 44 flanking deletions [17]. In BMD patients, mutant dystrophin levels have been shown to vary from <5% to 100% of a healthy control, with variable clinical phenotypes which don’t always correlate [13,30]. In line with this, in this study we found dystrophin levels ranging from 10% to 90% CTRL in a variety of 25 BMD samples. The majority of these BMD samples had been previously analysed by traditional WB using the dystrophin antibodies monoclonal NCL-DYS1 (epitope: mid rod domain) or C-terminal polyclonal ab15277 [13]. While the dystrophin levels detected by WB (C-terminal antibody: 3%-78%) were in a similar range to levels detected by Wes, the overall correlation was poor (R^2^ = 0.10), which is likely a combined consequence of differences in assay performance and the use of a different dystrophin antibody. The BMD samples with the lowest dystrophin levels (10% CTRL) were relatively close to the DMD samples with highest dystrophin (7% CTRL), which, being speculative, may suggest that dystrophin levels starting from 10% CTRL upwards would be the minimal amount to be aimed for in dystrophin-restoring therapies. It must however be noted that the skeletal muscle samples analysed here are only a relatively small subset, do not represent the full array of deletions in BMD and DMD, and more importantly were not selected to investigate the correlation between dystrophin levels and functional phenotype. In addition, the 31 healthy control samples we analysed were all derived from adults (age 22–75), and we cannot exclude that dystrophin levels in younger healthy male controls more similar in age to DMD boys participating in dystrophin-restoring clinical trials may be different to the spectrum of dystrophin levels characterised here in healthy control adults. In addition, different skeletal muscles may display differences in dystrophin levels, although we did not analyse sufficient numbers of the different skeletal muscle types to draw any conclusions on this. Having purified dystrophin protein available as a reference standard would be preferable. However, production of purified, stable full length dystrophin protein remains technically challenging and is not available at this time. The standard practice of expressing results as a percentage of a healthy control makes results easier to interpret, but may cause bias when comparing different studies, if different reference controls are used. We recommend to select a healthy control skeletal muscle sample as a reference standard that has mean dystrophin levels in a panel of healthy control samples and of which there is sufficient material available to allow it to be taken along in a large number of Wes analyses.

In conclusion, the Wes method for dystrophin detection is specific, sensitive and reproducible. It can detect trace dystrophin levels in DMD as well as the whole dystrophin range covered by BMD and healthy control muscle samples, and is thus suitable for quantification of dystrophin expression as a biomarker in DMD clinical drug development.

## Supporting information

S1 Fig Comparison of Wes ladder versus HiMark marker on traditional Western blot. 1: HiMark pre-stained marker (Invitrogen); 2: Wes ladder + streptavidin-IRDye800CW. The ladders do not completely line up in the high molecular weight region. The 440 band of Wes ladder migrated slower than 460 band of HiMark ladder, which can lead to underestimation of high molecular weights >280 kDa on the Wes.
(TIF)

S2 Fig Dynamic ranges for ab154168 and Mandys106 using 1.25 μg of healthy control sample spiked into a DMD sample. Both antibodies show good linearity from 0%-100% of healthy control when loading 1.25 μg of healthy control. Experiments were performed in triplicate.
(TIF)

S3 Fig Optimization of the anti-α-actinin antibody (ab16768). A) Antibody dilution response curve on 25 μg/ml healthy human muscle sample. B) Total protein loading response curve (healthy control), using 1/100 ab68167. The red arrows indicate the dilution/concentrations selected for further experiments.
(TIF)

S1 Table Overview of patient samples. (PDF)

S2 Table Antibody dilution and total protein loading concentrations selected for ab154168, Mandys106 and anti-α-actinin ab68167 (* = lowest tested). (PDF)
