# Correction: Use of capillary Western immunoassay (Wes) for quantification of dystrophin levels in skeletal muscle of healthy controls and individuals with Becker and Duchenne muscular dystrophy

**DOI:** 10.1371/journal.pone.0336651

**Published:** 2025-11-13

**Authors:** Chantal Beekman, Anneke A. Janson, Aabed Baghat, Judith C. van Deutekom, Nicole A. Datson

Contrary to the Data Availability statement in [1], the individual-level underlying data supporting the article’s results were not provided. The quantitative data underlying Figs 3-8, S2-S3, and Tables 2-3 are provided with this notice as S1–S10 Files.

## Supporting information

S1 File The original underlying quantitative data supporting Figure 3. (XLSX)

S2 File The original underlying quantitative data supporting Figure 4. (XLSX)

S3 File The original underlying quantitative data supporting Figure 5, panel B. (XLSX)

S4 File The original underlying quantitative data supporting Figure 6, panel A. (XLSX)

S5 File The original underlying quantitative data supporting Figure 7. (XLSX)

S6 File The original underlying quantitative data supporting Figure 8. (XLSX)

S7 File The original underlying quantitative data supporting Table 2. (XLSX)

S8 File The original underlying quantitative data supporting Table 3. (ZIP)

S9 File The original underlying quantitative data supporting Figure S2. (XLSX)

S10 File The original underlying quantitative data supporting Figure S3. (ZIP)
