# Supplementary figures and images for: Use of capillary Western immunoassay (Wes) for quantification of dystrophin levels in skeletal muscle of healthy controls and individuals with Becker and Duchenne muscular dystrophy
Source: PLoS One. 2018 Apr 11;13(4):e0195850. doi: 10.1371/journal.pone.0195850 (PMC5895072; doi:10.1371/journal.pone.0195850)

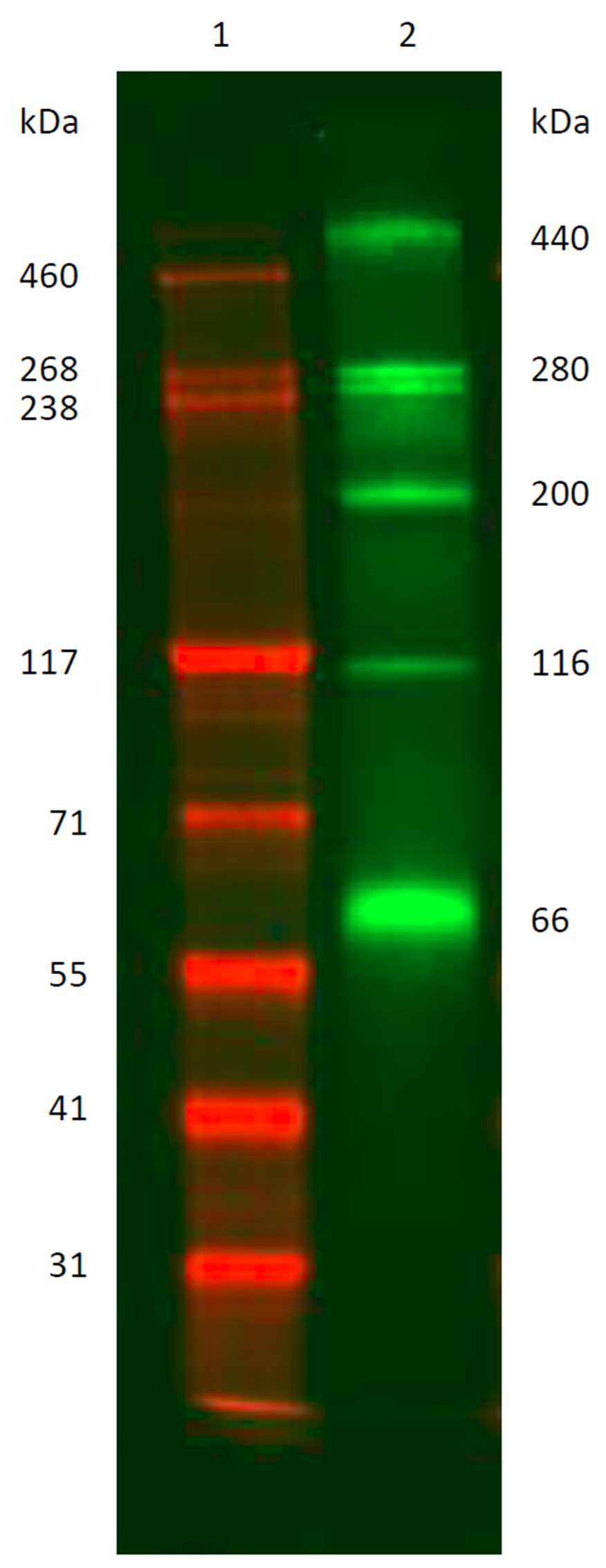

Supplement: S1 Fig — 1: HiMark pre-stained marker (Invitrogen); 2: Wes ladder + streptavidin-IRDye800CW. The ladders do not completely line up in the high molecular weight region. The 440 band of Wes ladder migrated slower than 460 band of HiMark ladder, which can lead to underestimation of high molecular weights >280 kDa on the Wes. (TIF) [file pone.0195850.s001.tif]

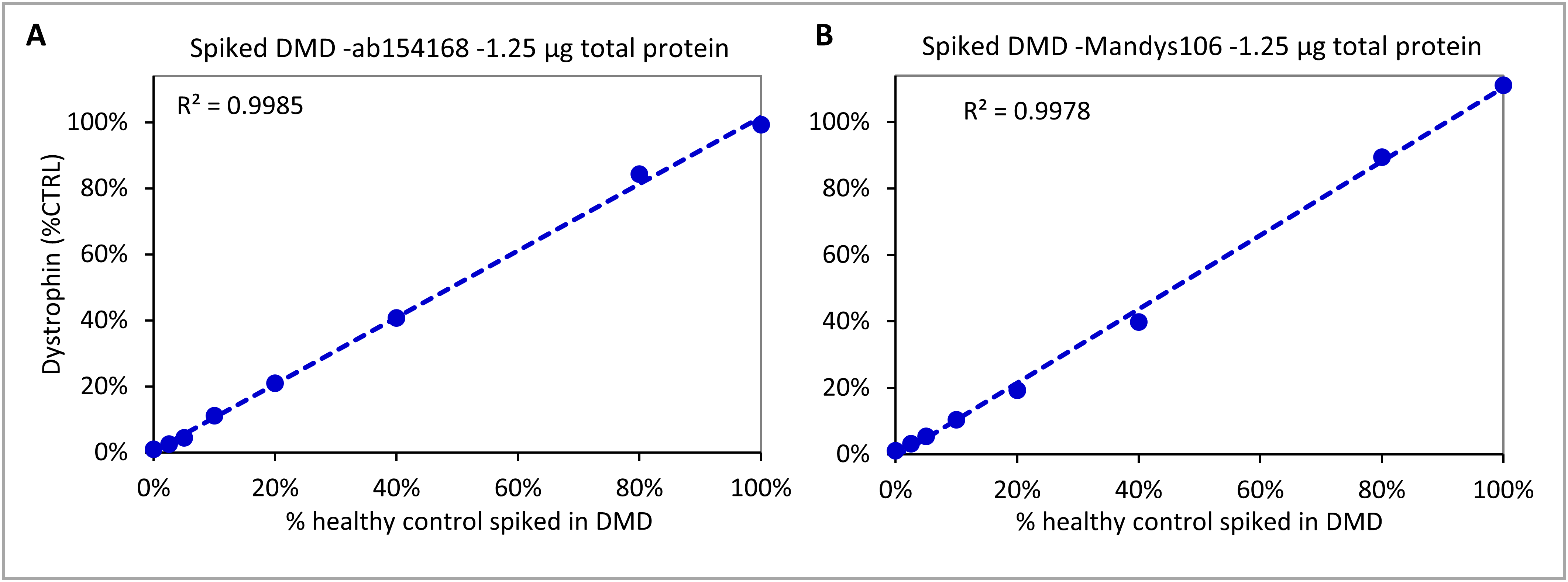

Supplement: S2 Fig — Both antibodies show good linearity from 0%-100% of healthy control when loading 1.25 μg of healthy control. Experiments were performed in triplicate. (TIF) [file pone.0195850.s002.tif]

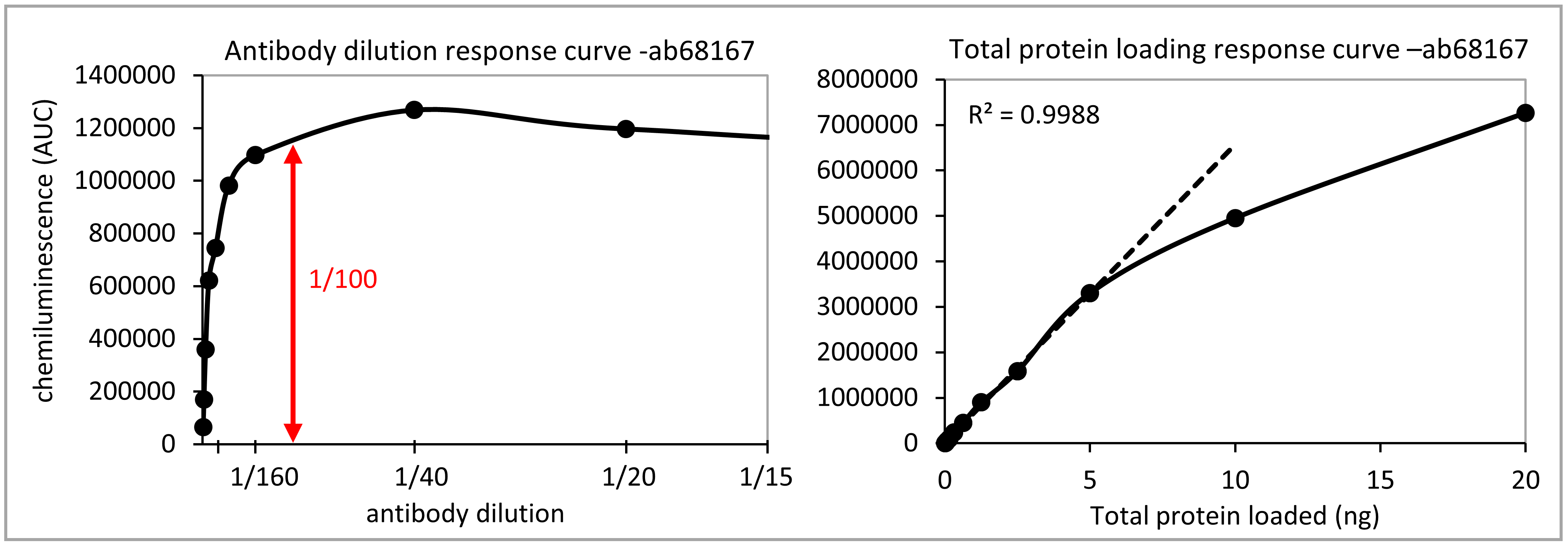

Supplement: S3 Fig — A) Antibody dilution response curve on 25 μg/ml healthy human muscle sample. B) Total protein loading response curve (healthy control), using 1/100 ab68167. The red arrows indicate the dilution/concentrations selected for further experiments. (TIF) [file pone.0195850.s003.tif]
