# Supplementary material for: Use of capillary Western immunoassay (Wes) for quantification of dystrophin levels in skeletal muscle of healthy controls and individuals with Becker and Duchenne muscular dystrophy
Source: PLoS One. 2018 Apr 11;13(4):e0195850. doi: 10.1371/journal.pone.0195850 (PMC5895072; doi:10.1371/journal.pone.0195850)
Supplement: S1 Table — (PDF) [file pone.0195850.s004.pdf]

Healthy control samples				BMD and DMD samples			
**Sample**	**Gender**	**Age**	**Muscle**	**Sample**	**Muscle**	**Age**	**Deletion/Mutation**
Control0*	male	~50	Tibialis	BMD1	Tibialis	56	unknown
Control1	unknown	~75	Quadriceps	BMD2	Tibialis	47	del. 45-47
Control2	unknown	~75	Quadriceps	BMD3	Tibialis	48	del. 45-48
Control3	unknown	~75	Quadriceps	BMD4	Tibialis	29	Ex.19:c.2380+3A>C
Control4	unknown	~75	Quadriceps	BMD5	Tibialis	51	del. 45-55
Control5	male	37	Quadriceps	BMD6	Tibialis	19	del. 05
Control6	male	~50	Quadriceps	BMD7	Tibialis	47	del. 10-22
Control7	male	~50	Quadriceps	BMD8	Tibialis	30	Ex.26:c.3515G>A,p.Trp1172X
Control8	male	~50	Quadriceps	BMD9	Tibialis	26	del. 45-47
Control9	male	~50	Quadriceps	BMD10	Tibialis	unknown	Ex.29:c.3940C>T,p.Arg1314X
Control10	male	67	Quadriceps	BMD11	Tibialis	63	del. 45-47
Control11	female	48	Quadriceps	BMD12	Tibialis	57	del. 03-07
Control12	male	53	Quadriceps	BMD13	Tibialis	47	del. 45-47
Control13	male	53	Quadriceps	BMD14	Tibialis	33	del. 03-05
Control14	male	47	Quadriceps	BMD15	Tibialis	39	del. 45-47
Control15	male	47	Quadriceps	BMD16	Tibialis	unknown	unknown
Control16	unknown	61	Tibialis	BMD17	Tibialis	37	del. 45-47
Control17	male	~50	Tibialis	BMD18	Tibialis	51	unknown
Control18	male	~50	Tibialis	BMD19	Tibialis	20	del. 45-47
Control19	male	69	Tibialis	BMD20	Tibialis	31	del. 03-04
Control20	male	36	Tibialis	BMD21	Tibialis	29	del. 45-47
Control21	male	49	Tibialis	BMD22	Tibialis	unknown	del. 48-49
Control22	male	52	Tibialis	BMD23	Tibialis	39	del. 45-47
Control23	male	57	Tibialis	BMD24	Tibialis	51	del.45-47
Control24	female	39	Tibialis	BMD25	Tibialis	31	del. 45-47
Control25	male	51	Tibialis	DMD1**	Biceps	10	del. 45
Control26	female	36	Tibialis	DMD2	Biceps	13	del. 45-54
Control27	male	30	Tibialis	DMD3	Biceps	10	del. 14-43 [##]
Control28	female	28	Tibialis	DMD4	Biceps	14	del. 43 [##]
Control29	female	33	Tibialis	DMD5	Gastrocnemius	9	del. 46-48
Control30	male	66	Tibialis	DMD6	Gastrocnemius	11	del. 44
Control31	male	22	Tibialis	DMD7	Gastrocnemius	8	del. 12-44 [##]
				DMD8	Gastrocnemius	8	del. 46-53
				DMD9	Gastrocnemius	14	del. 45-52
				DMD10[#]	Gastrocnemius	10	del. 48-52
				DMD11	Gastrocnemius	7	del. 45-52
				DMD12	Gastrocnemius	6	del. 52
				DMD13	paravertebral	15	del. 45
				DMD14	unknown	16	del. 45-50
				DMD15	Tibialis	4	del. 46-49
				DMD16	Tibialis	7	del. 52-54
				DMD17[$]	Tibialis	11	del. 46-49

* healthy reference control (used for calibration curves and spiking)
** 'DMD high' in validation experiments
# 'DMD low' in validation experiments
$ DMD used for spiking experiments
lacks Mandys106 epitope
