# Supplementary material for: Use of capillary Western immunoassay (Wes) for quantification of dystrophin levels in skeletal muscle of healthy controls and individuals with Becker and Duchenne muscular dystrophy
Source: PLoS One. 2018 Apr 11;13(4):e0195850. doi: 10.1371/journal.pone.0195850 (PMC5895072; doi:10.1371/journal.pone.0195850)
Supplement: S2 Table — (PDF) [file pone.0195850.s005.pdf]

**antibody**	**Optimal antibody dilμtion**	**Linear range loading conc. healthy control**	**Linear range loading conc DMD**	**Selected standard loading concentration**
ab154168	1/1000<br>(0.28 μg/ml)	0.2 - 100 μg/ml<br>(500-fold)	31.3 – 300 μg/ml	25 μg/ml (healthy control)<br>250 μg/ml (DMD)
Mandys106	1/50<br>(20 μg/ml)	3.1 - 200 μg/ml<br>(60-fold)	31.3 – 400 μg/ml	25 μg/ml (healthy control)<br>250 μg/ml (DMD)
α-actinin	1/100<br>(14.2 μg/ml)	0 - 5 μg/ml (healthy control & DMD)		2.5 μg/ml<br>(healthy control & DMD)
